# Body mass index has an impact on preoperative symptoms but not clinical outcome in acute cauda equina syndrome

**DOI:** 10.1038/s41598-021-92969-4

**Published:** 2021-07-06

**Authors:** Vicki M. Butenschoen, Shadi Abulhala, Bernhard Meyer, Jens Gempt

**Affiliations:** Department of Neurosurgery, School of Medicine, Klinikum Rechts der Isar, Technical University of Munich, Ismaningerstr. 22, 81675 Munich, Germany

**Keywords:** Spinal cord diseases, Neurophysiology

## Abstract

Cauda equina syndrome (CES) presents a surgical emergency with treatment required within 48 h. Symptoms include reduced saddle sensation, micturition difficulties, and/or anal sphincter impairment. Controversy exists regarding the effect on and coincidence of overweight with CES. We performed a retrospective case–control study of all patients treated surgically for acute complete and incomplete CES in our neurosurgical department from 2009 to 2020, focusing on the preoperative BMI and postoperative neurological outcome. In addition, we performed a comprehensive literature review. Fifty patients with CES were included, of whom 96% suffered from a decompensated lumbar spinal stenosis or disc prolapse between the L4/5 and L5/S1 levels. Our cohort population was overweight but not obese: mean BMI was 27.5 kg/m^2^, compared with 27.6 kg/m^2^ in patients with degenerative spine surgery. BMI did not significantly influence the postoperative outcome, but it did affect preoperative symptoms and surgery duration. Symptom duration significantly differed depending on the underlying cause for CES. The literature review revealed sparse evidence, with only four clinical case series presenting contradictory results. We provide a comprehensive literature review on the current evidence regarding CES and obesity and conclude that we did not observe an association between obesity and CES occurrence. Patients with CES and other degenerative spinal pathologies belong to an overweight but not obese population. Body Mass Index has an impact on preoperative symptoms but not clinical outcome in acute CES.

## Introduction

Patients with acute cauda equine syndrome (CES) present with sensation loss of the perineal region, urinary retention, or loss of anal sphincter control. Even though these conditions are rare, patients are significantly burdened due to the limited recovery rates and persistent impaired vegetative functions.

Most patients presenting with acute symptoms of cauda equina compression suffer from extensive disc prolapses compressing the cauda equina nerve roots^1,2^ or show evidence of a decompensated spinal canal obstruction^3^. Treatment should be performed within a short time margin and requires emergency magnetic resonance imaging (MRI) to confirm the diagnosis, followed by prompt surgical decompression^3–5^.

The coincidence of an increased body mass index (BMI) and CES has been discussed as one of the risk factors for CES occurrence, especially in young patients.

Reviewing the literature, only 10 studies addressed the controversy over BMI and CES, of which 4 included retrospective (n = 3) or prospective (n = 1) clinical studies, and 6 publications were only single case reports. Of the 4 clinical studies, 2 concluded that an increased BMI significantly increased the risk for CES (Cushnie et al.^6^: 31.8 vs. 28.1 mg/m^2^, *p* = 0.007; Venkatesan et al.^7^: odds ratio [OR] 1.17, *p* < 0.001), while 2 denied a significant association between CES and increased BMI (Shen et al.^8^: BMI of CES patients 27.5 kg/m^2^; Kaiser et al.^9^). All studies were classified as level III and level IV studies (following the Oxford Centre for Evidence-Based Medicine [OCEBM] levels of evidence).

Due to this lack of evidence and sparse literature, we aimed to review our own experience in a retrospective monocentric case–control study to explore past and present controversies over increased weight and CES.

## Methods

### Study cohort

We conducted a retrospective monocentric study of all consecutive patients treated between January 2009 and June 2020, including all patients who underwent operation due to acute CES, as well as available data on weight, size, and comorbidities.

Pre- and postoperative data (with description of the surgical treatment and affected spinal level), as well as preoperative imaging, were retrieved from our records. Clinical information included the preoperative BMI, the presence of comorbidities or previous neurosurgical treatment, and the duration of symptoms. The clinical state before and after surgery and during follow-up was assessed, and the occurrence of CES was defined as incomplete if only one or two of the following symptoms were present: bladder dysfunction with urinary retention, reduced or missing anal sphincter tonus, and genital sensory disturbance. If all symptoms were present, CES was classified as complete.

### Study design

We conducted a retrospective monocentric analysis in a high-volume neurosurgical center. We assessed relevant details leading to the indication of the surgical intervention, intraoperative findings, and procedure, and we reviewed follow-up data to assess the recovery rate. To compare the BMI of our sample with that of a control population, we retrieved data on weight and height from 50 age-controlled patients without symptoms of CES but matching spine pathologies such as lumbar spinal stenosis and disc herniation. All patients underwent emergency surgery with spinal decompression and sequestrectomy if necessary. Surgery was performed within 24 h in all patients suffering from CES after presenting in our neurosurgical emergency department.

### Statistics

Statistical analyses were performed using SPSS Statistics 26 (IBM, Chicago, IL). Binomial dichotomized data were compared using Fisher’s exact test, and categorical data were compared using the chi-squared test.

Median or mean values were compared using a Student’s *t* test when appropriate. The association between potential factors and postoperative outcome (using follow-up data or discharge data for those with a missing follow-up) was analyzed using ANOVA. The following factors were assumed as potentially predictive: BMI, symptom duration, entity, surgery duration, age, and sex. We compared patients suffering from a decompensated spinal canal obstruction with patients suffering from soft disc prolapses. To compare weight, height, and BMI, we analyzed the data of 50 age-adjusted patients suffering from the same spinal pathologies without symptoms of CES (control group).

*P* values less than 0.05 were considered statistically significant.

### Ethics and consent to participate

The research conducted has been performed in accordance with the Declaration of Helsinki. Also, we obtained a positive vote by a local ethics committee (Prof. Dr. Georg Schmidt, Technical University Munich Ethics Commission).

### Consent for participation and publication

Due to the retrospective nature of the study, prospective patient consent was not required and waived by the local ethics committee.

## Results

### CES patient population

In total, 50 consecutive patients were identified and included for statistical analysis. All presented with symptoms of complete (20/50, 40%) or incomplete (30/50, 60%) CES and underwent immediate surgical treatment within 24 h. Of the patients, 28/50 (56%) were male and 22 (44%) were female. One patient was pregnant during surgery (gestational age 10 weeks).

Median age was 42 years (range 19 to 83, IQ 32–66 years). Median age significantly depended on the underlying pathology (median age in patients with disc prolapses 40 years vs. decompensated lumbar stenosis 71 years, p < 0.001).

Of the patients, 22% (11/50) had a prior history of surgical treatment at the same (10/11) or adjacent spinal level (1/11). In 84% of the patients, the pathology was mono-segmental (42/50), and in 16% (8/50) of the cases, the patients underwent a decompression of 2 (6/8) or more (2/8) lumbar spinal segments. The most affected levels were L5/S1 (52%, 26/50 cases) and L4/L5 (44%, 22/50 cases).

Eighteen patients presented with motor deficits of the lower extremities (36%) ranging from Medical Research Council (MRC) 2/5 to 4/5, including plantar and dorsiflexion of the foot, but also knee extension and hip flexion.

**Figure 1:**
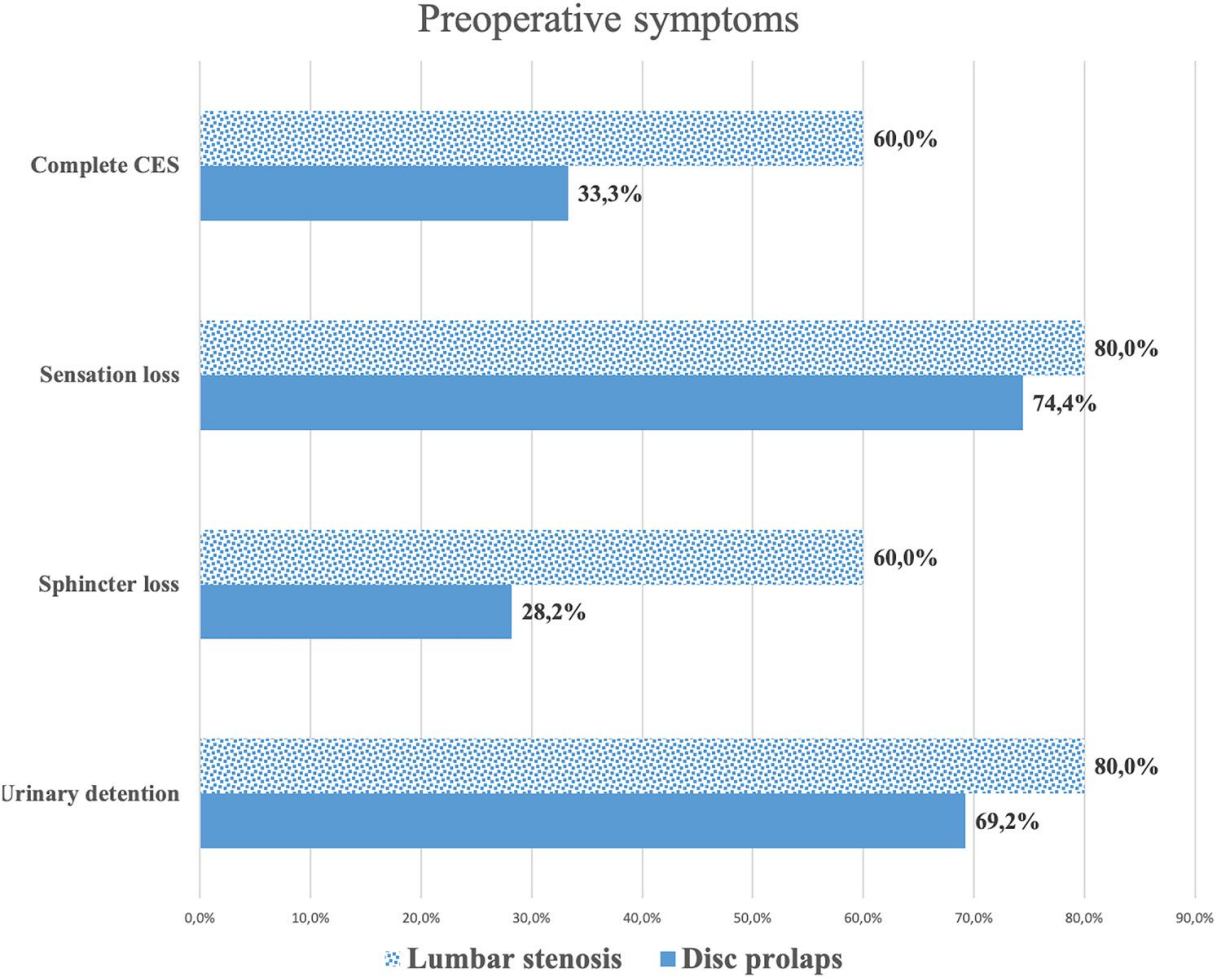
Preoperative symptoms described by patients with complete and incomplete cauda equina syndrome (CES).

Urinary retention was described by 72% of the patients, perineal hypesthesia in 76%, and anal sphincter tone reduction in 36% (Fig. 1).

Thirty-nine patients (78%) suffered from giant lumbar disc prolapses occupying more than 40% of the spinal canal, 10 patients (20%) from a central spinal canal stenosis, and 1 (2%) patient from an epidural hematoma due to phenprocoumon overdose.

Median symptom duration before surgery was 2 days (range 0 to 80 days, IQ 1–4 days), with patients suffering from spinal canal stenosis having a longer symptom duration than patients with disc prolapses, without reaching statistical significance (mean 5.4 vs. 2.8 days, p = 0.357).

### Weight and its influence on outcome

Mean preoperative weight was 85.2 kg (range 50 to 170 kg, IQ 70–90 kg, compared with control group: mean weight 84.7 kg, p = 0.926), and mean height was 1.75 m (range 1.58 to 1.98 m, IQ 1.69–1.85 m, compared with control group: mean height 1.74 m, p = 0.772). Mean BMI showed an overweight but not obese patient population: 27.5 kg/m^2^ (range 19.2 to 55.5 kg/m^2^, IQ 23.5–30.5 kg/m^2^). We did not detect any significant difference in the BMI between patients with decompensated lumbar spinal stenosis and patients with disc prolapses (BMI 27.9 vs. 27.5 kg/m^2^, p = 0.91) (Table 1). Compared with our control group of patients without symptoms of CES operated on for degenerative spine pathologies, mean BMI for patients with CES showed no significant difference (27.5 vs. 27.6 kg/m^2^ for patients with and without CES, respectively, p = 0.959) (Table 2). Classified in different BMI categories, 23/50 patients presented with a normal weight (BMI 18.5–24.9 kg/m^2^), 14/50 patients belonged to an overweight population (BMI 25–29.9 kg/m^2^), and 13/50 patients belonged to an obese population (BMI > 30 kg/m^2^).

**Table 1:** Characteristics of patients suffering from cauda equina syndrome (CES), subgrouped by underlying cause of CES showing significant differences (*) in age and direct postoperative recovery.

	Lumbar stenosis	Disc prolapse	Total	p-value
Median age (years)	71.5	40	41.5	0.000*
Complete CES	60%	33.3%	40%	0.143
Mean symptom duration	5.4 days	2.8 days	3.3 days	0.357
Previous surgery (%)	10%	25.6%	22%	0.491
**Postoperative recovery**				0.002*
Complete	20%	23.1%	22%	
Incomplete	80%	71.8%	72%	
None	0%	5.1%	6%	
Recovery at follow-up	85.7%	84.2%	81.5%	0.713
Mean BMI (kg/m^2^)	27.9	27.5	27.5	0.910

**Table 2:** Case–control data to compare the population with cauda equina syndrome (CES) to an age-adjusted cohort of patients undergoing spinal surgery for other degenerative spinal pathologies.

	CES	No CES	P value
Age (mean years)	48.1	49.2	0.777
Female sex	51%	44%	0.552
Height (mean)	1.75 m	1.74 m	0.772
Weight (mean)	85.2 kg	84.7 kg	0.926
BMI (mean)	27.5	27.6	0.959

Analyzing preoperative symptoms in different BMI categories, patients suffered significantly more often from preoperative urinary detention in the obese group (100%) compared with the normal (60.9%) and overweight (64.3%) groups (p = 0.032). A similar tendency was observed for saddle sensation loss, but this failed to reach statistical significance. Although it was diagnosed in 100% of the obese population, sensation loss was reported in 65.2% of the patients categorized as normal weight and 71.4% in the overweight population (p = 0.052). The occurrence of motor deficits did not significantly differ between all groups.

Comparing the clinical outcome of patients subgrouped in different BMI categories, the improvements did not depend on the weight category (p = 0.616 for improvement at follow-up, p = 0.464 for postoperative improvement).

### Surgical procedure

Mean duration of surgery was 78 min (range 32 to 213 min, IQ 52–99 min) and significantly depended on the BMI category to which the patient belonged (normal BMI: 67 min, overweight: 78 min, obese patients: 97 min, *p* = 0.042). Patients operated on for lumbar spinal stenosis underwent longer surgeries, but the difference failed to reach statistical significance (mean surgery duration 96 vs. 73 min, *p* = 0.168). None of the patients needed instrumentation. No intraoperative complications occurred.

### Spinal canal stenosis vs. disc prolapse

The underlying entity causing CES significantly influenced the direct postoperative outcome: Patients with disc prolapses recovered more frequently than did patients with lumbar stenosis. In the population suffering from disc prolapse, complete recovery occurred in 9/39 patients and incomplete recovery in 28/39 patients, vs. 2/10 and 8/10, respectively, in patients suffering from decompensated spinal canal stenosis (Chi^2^, *p* = 0.002). The preoperative BMI did not influence the clinical outcome (*p* = 0.111).

The risk for permanent bladder dysfunction was higher in the spinal canal stenosis group than in patients undergoing surgery for a lumbar disc herniation and CES (10% vs. 7.7%, *p* = 0.01) but did not depend on the number of segments operated upon or a patient’s BMI (*p* = 0.123 and *p* = 0.885). Motor deficits improved in 23.1% of the patients suffering from disc herniation and in 20% of the spinal stenosis patients, but the difference was not significant (*p* = 0.425).

Median length of hospital stay (LOH) was 4 days and significantly depended on the underlying cause of CES (lumbar spinal stenosis 7.5 days vs. disc prolapse 4 days, *p* = 0.005) but not on the preoperative BMI (*p* = 0.524).

### Postoperative complications and neurological outcome

In total, 2/50 patients suffered from a postoperative hemorrhage causing radiating pain without new neurological deficits (4%). One patient did not recover after surgery. A postoperatively performed MRI revealed a persistent compression due to residual prolapse tissue, and the patient underwent a second surgery to remove the disc prolapse, resulting in a surgical revision rate of 6%. The 30-day mortality was 0%.

Overall, 47/50 patients reported an improved clinical status after surgery until discharge (94%) after a median of 4 days: In 36/47 patients (76.6%), we assessed an improvement of preoperative symptoms with residual saddle hypoesthesia or residual urinary detention, and in 11/47 patients (23.4%), the preoperative CES resolved completely. In 4% of the patients (2/50), the postoperative symptoms of CES remained unchanged (2/3), and one patient suffered from neurological deterioration without radiographical correlate (2%).

Treatment for recurrent disc prolapse did not influence the recovery rate (improvement of preoperative symptoms in 11/11 vs. 36/39 patients, *p* = 0.271), nor did age (*p* = 0.059).

In total, long-term follow-up was available in 27/50 patients (54%) after a median follow-up time of 114 days (range 38–1825 days).

Of the patients, 22/27 (81.5%) reported further relief from preoperative symptoms, and five patients remained stable.

At follow-up, one third of the patients (9/27, 33.3%) described an improvement compared with the preoperative symptoms but still suffered from perineal sensation impairment. Improvement at follow-up significantly depended on the presence of a complete vs. incomplete CES (63.6% improvement in complete CES vs. 93.8% in incomplete CES, *p* = 0.048) but not on the underlying entity causing the CES (*p* = 0.101). The rate of clinical recovery and persistence of numbness is congruent with published literature, describing better results in patients suffering from incomplete CES compared with complete CES^10^. BMI was not significantly associated with the probability of neurological recovery (mean BMI in patients with complete recovery 26.8 vs. 28.4 kg/m^2^, *p* = 0.577); symptom duration differed but failed to reach statistical significance (mean symptom duration in patients with complete recovery 2.6 days vs. 7.8 days in patients without complete recovery, *p* = 0.086).

## Discussion

### Overweight patient population in spinal surgery

In our study, the patient population was overweight but not obese (BMI 27.5 kg/m^2^, overweight: BMI 25–30, obesity: BMI > 30 kg/m^2^). Compared with the BMI of patients undergoing other surgeries such as lumbar decompression surgery due to spinal canal stenosis without CES, the described BMI ranges around 29.4 kg/m^2^ in current literature^11^. We therefore support the hypothesis of Cushnie et al. and Venkatesan et al.: In our cohort study, CES was not associated with obesity, but patients undergoing spinal lumbar surgery seemed to belong to an overweight population, similar to our control patients undergoing spinal surgery for degenerative spinal pathologies without exhibiting symptoms of CES. The patient population for CES and other spinal degenerative pathologies seems to be overweight in general. Although complications in overweight patients are described to be higher, satisfying results can be achieved^12^.

### Spinal epidural lipomatosis

Most published case reports address the problems of spinal lipomatosis, obesity, and CES^13^. Although the idea seems obvious, a study by Alicioglu et al. in 2008 did not support the correlation of spinal epidural fatty tissue with BMI and abdominal obesity^14^. In our study, we did not assess the amount of fatty tissue in the spinal canal—therefore, a statement regarding lipomatosis and CES cannot be made from our retrospective cohort population.

### Limitations

Our clinical cohort study presents retrospective data from a single high-volume institution. A prospective enrolment of obese patients and patients of normal weight would increase the evidence level, but up to now, the only prospective study, performed by Kaiser et al.^9^ with 35 prospectively enrolled patients, did not support the coincidence of CES and obesity.

We did compare the assessed patients with other patients operated on for degenerative diseases of the lumbar spine, and the results support the current literature on BMI and spinal surgery describing an overweight population in general. We only included patients suffering from a degenerative spinal pathology; therefore, we cannot draw any conclusions on patients suffering from other spinal pathologies, such as tumor lesions or spinal trauma.

Unfortunately, follow-up data was available in only 54% of the cases, as patients who underwent surgery for degenerative spine disease do not follow a regular outpatient control appointment. The low rate of patients available for follow-up presents a strong limitation of our study, but complete recovery after surgery was observed, allowing us to hope that patients continued to improve after hospital discharge. Only three patients did not benefit from the surgical treatment; therefore, statistical analysis may underestimate the prognostic value of different parameters such as age, underlying cause of CES, and BMI. Furthermore, sexual dysfunction was often neglected in the clinical assessment before and after surgery, and the collected data are too sparse to include it for further analysis. Poor documentation on sexual function often occurs when assessing cauda equina symptoms, and this limitation has been described in current literature^15^.

Comparing CES patients with decompensated spinal canal stenosis and soft disc prolapse patients, we importantly identified significant differences in median age, symptom duration, and neurological recovery. As symptom duration influences neurological recovery, patients presenting with decompensated spinal canal stenosis should be examined for CES symptoms appropriately. To our knowledge, subgroup analyses in CES patients focusing on the differences in their neurological outcome depending on the underlying cause of surgical intervention have not been described before. We hereby present novel data useful for every surgeon advising a patient suffering from CES regarding clinical outcome after surgery. We provide a distinct subgroup analysis useful to address the expectations of CES patients, whether they present with large disc herniation or decompensated lumbar stenosis.

Reviewing the literature on CES and increased patient weight, we identified only 4 clinical studies investigating the subject of obesity and CES. All studies were classified as level III to IV evidence, and they tended to be descriptive cohort studies, most of which lacked a control group.

## Conclusion

We hereby present a large cohort study of patients suffering from acute CES with a special focus on weight, CES occurrence, and postoperative outcome, and we compare those parameters depending on the underlying cause for CES. We did not observe an association between obesity and CES occurrence, patients with CES and other degenerative spinal pathologies belong to an overweight but not obese population. In our study, BMI had an impact on preoperative symptoms but not clinical outcome in acute CES.

## Data Availability

The datasets used and/or analyzed during the current study available from the corresponding author on reasonable request.
